# Measuring impacts: a scoping review of healthcare impact evaluations

**DOI:** 10.1186/s12961-025-01324-w

**Published:** 2025-09-11

**Authors:** L. R. Correia, J. C. Martins, E. T. Rother, P. C. de Soárez

**Affiliations:** 1 Departamento de Medicina Preventiva da Faculdade de Medicina da, Universidade de São Paulo, São Paulo, Brazil; 2 Hospital Israelita Albert Einstein, São Paulo, Brazil; 3 Brasilia, Brazil

**Keywords:** Impact evaluation, Scoping review, Healthcare interventions

## Abstract

**Background:**

With the increasing use of the term “impact evaluation” in healthcare and the absence of an exhaustive review on this general theme, this research aims to map available evidence and methods associated with impact evaluations in healthcare by conducting a scoping review.

**Methods:**

This exhaustive review included peer-reviewed studies of healthcare interventions with no restrictions on language or time of publication.

**Results:**

In total, 324 studies met the inclusion criteria from 4372 single registries retrieved from Medline, Embase, Scopus, WoS and Econlit in August 2024, with no time restriction. Only ex-post studies were identified; as expected from guidelines, most studies used counterfactuals (58%) and only 7% did not use any comparison. Furthermore, natural experiments or quasi-experiments were the most applied designs (37%), followed by observational (26%) and experimental (17%) designs.

**Conclusions:**

Impact evaluations of healthcare interventions seem to be predominantly associated with methods of strong comparison (counterfactuals) designs as seen in guidelines; however, there are exceptions.

**Supplementary Information:**

The online version contains supplementary material available at 10.1186/s12961-025-01324-w.

## Introduction

Health systems suffer financial pressures worldwide, and evaluations are valuable tools to help decision-making amid this scenario [1]. Specifically, impact evaluation has been studied in many ways, for example, a recent study by Vigneri analysed similarities and differences of these methods in the context of epidemiology and development economics [2].

Although there are exhaustive reviews on impact evaluation of specific healthcare interventions, such as Degroote’s “Health insurance in Sub-Saharan Africa: a scoping review of the methods used to evaluate its impact” [3], as far as the authors of this article know, there has been no exhaustive review mapping the use of impact evaluation as a concept when evaluating general healthcare interventions. This research intends to fill this gap with a scoping review which is an evidence synthesis methodology that maps relevant publications according to established criteria [4].

As stated in the registered protocol [5], the primary research question is “What methodologies and approaches are used in impact evaluations in the context of healthcare project evaluation?” A secondary research question is “What is the evidence available on impact evaluation in healthcare projects?”

Typically, guidelines are used to help develop these evaluations. In particular, a few guidelines on impact evaluation are available, with many healthcare examples. Gertler is commonly used for interventions in various subjects [6]. Specifically, in health, Clarke provides a general view [7], and a few countries have their own guidelines, such as the one recently created by the Ministry of Health in Brazil [8].

This article assumes a few definitions from these guidelines. The concept of impact evaluation is an inquiry into the causality of an intervention to any specific outcome – healthcare intervention as any human-made action intended to promote positive health outcomes to a group of people, including policies, programs, projects, among others, and is related to various health conditions. Another key definition is a counterfactual to identify this causality, that is, what would have happened to the population that received the intervention had they not received it [7], which from these guidelines is quantitative evidence. Furthermore, quasi-experiments or natural experiments are designs that use quasi-randomization to perceive counterfactuals and bring rigor to observational studies [9].

In Colchero’s article, “A systematic review of the literature on the impact of the Seguro Popular”, these evaluations with counterfactuals are specified as rigorous, and the review focuses on them [10]. That is because, as observed further on, many studies identify themselves as impact evaluations but do not completely follow the characteristics of a counterfactual mentioned in the guidelines.

Thus, the goal of this research is a scoping review to map peer-reviewed literature on the theme. It investigates the methodologies used in impact evaluations of healthcare interventions as well as the available evidence on this theme.

## Methods

To achieve the aim of mapping the literature of impact evaluation in health care, this research is a scoping review based on Prisma-Scr as well as on the Joanna Briggs Institute (JBI) guidelines [11, 12]. The protocol for this research is registered at OSF: https://osf.io/453wp [5].

### Information sources and search strategy

The publications were retrieved from five databases: MEDLINE (Pub Med); Embase; Scopus; Web of Science; and EconLit. The authors of this review chose these after a thorough discussion proposed by a librarian, to best map the articles on the theme, considering the healthcare focus, as well as by researchers that typically conduct impact evaluations, such as economists. Owing to the focus on peer-reviewed studies, no grey literature was used.

The search strategy employed considered only the exact term “impact evaluation” as keywords within the text, aiming to prevent the omission of articles with varying approaches to health intervention. This decision was based on a pilot strategy that utilized general health terms and Boolean operators, which demonstrated a significant reduction in the number of results that would affect the quality of the review.

Albeit simple, this strategy was thoroughly developed with E.T.R., an experienced librarian, and the full electronic search strategies for all the five databases are in Additional File 1 and the paragraph below. The search took place in August 2023, with no date limitation, and was updated in August 2024, including studies from August 2023 to August 2024.

Search strategies: PubMed/Medline – “Impact evaluation” [TW]; Embase – “Impact evaluation” ['ti,ab,kw]; Scopus – TITLE-ABS-KEY (“Impact evaluation”) AND LIMIT-TO EXACTKEYWORD, (“Impact evaluation”); Web of Science – “Impact evaluation” (Topic) AND Article (Document Types) AND Science Citation Index Expanded (SCI-EXPANDED) (Web of Science Index) AND Article (Document Types); Ecolint – (“Impact Evaluation”) free text.

### Selection process and inclusion criteria

The selection was conducted in two phases: screening of titles and abstracts was performed by two researchers independently, and conflicts were solved by a third one; the selection of full articles was carried out by the two researchers independently, and conflicts were solved by consensus.

The inclusion criteria were the following: peer-reviewed studies that identified themselves as impact evaluations, therefore excluding conference abstracts and protocols or studies using similar terms, such as “impact assessments”. The focus of the studies had to be on human interventions in healthcare, thus excluding studies of nature-made interventions (such as a pandemic), or on non-health-related outcomes, such as criminality or nutrients by themselves. There was no restriction on study design or year of publication or language; papers written in languages other than English, Spanish, French or Portuguese were translated with Google Translate [13]. Additional File 1 includes these inclusion and exclusion criteria.

### Data extraction and analysis

For data extraction, the software Endnote – X9 version was used; as for the selection process, the online version of Rayyan software was adopted. Finally, Excel^®^ was used to collect the data and elaborate tables and charts. Duplicated studies were removed in three phases: with Endnote; Rayyan software; and a few left (10 registries) during screening (most of the former were from the update in August 2024).

The data items collected from each study that was included were title of publication; authors; year of publication; publisher; language; country where intervention took place; country of institution first authors were affiliated with; whether the study was ex-ant or ex-post (both reported and observed); intervention name and description; study methods and designs (including comparison level); sample; summary of results (including if positive or negative); and conflict of interests (reported and observed). Specifically, the comparison level, publication bias (direction of results) and conflict of interest variables are intended to answer the research question on the basis of the evidence available. A more detailed list of extraction data items is provided in Additional File 1.

The interventions were categorized into six types according to how they were perceived: (1) management, as to directly intervening in the management of governments, hospitals, among others; (2) assistance, as directly intervening in vulnerable people with immediate need; (3) education, including all teaching and learning programs and projects; (4) promotion, including preventive campaigns and communications; (5) research, intended to alleviate any health issue; and (6) pharmacologic, any direct medication or vaccination intervention.

Methods were categorized into three levels, as to whether they were qualitative, quantitative, used mixed-methods or were reviews. Within the quantitative ones, as to their study designs, they were mapped as: experimental, observational, quasi-experimental (or natural experiment) and mathematical modelling; finally, the quasi-experimental methods were mapped as to specific designs used.

The risk of bias and other evidence assessments were not performed as these do not apply to scoping reviews [11, 12].

## Results

### Characteristics of the studies

**Fig. 1:**
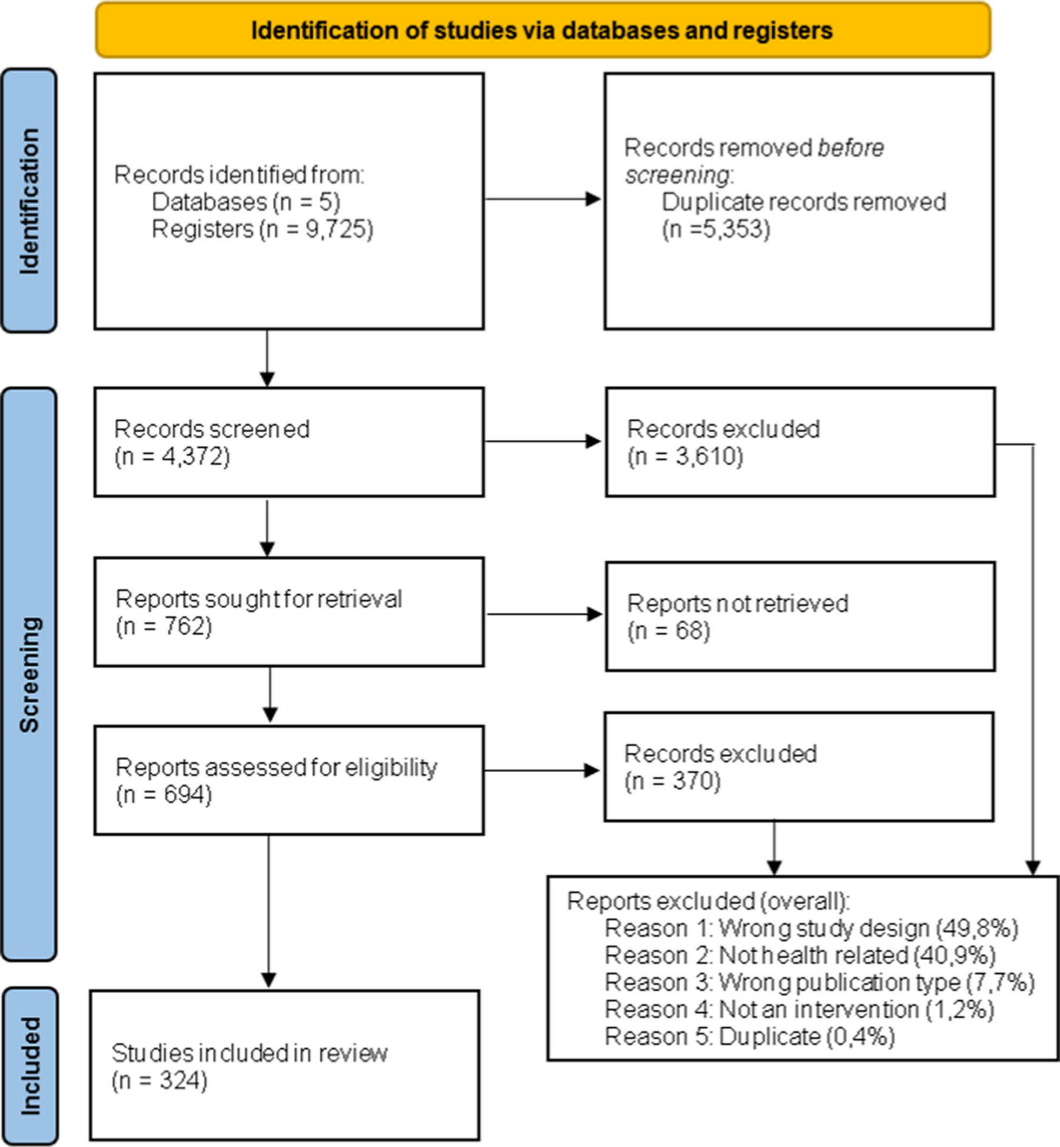
Flow diagram of scoping review of impact evaluations of healthcare interventions

The flowchart of this scoping review is presented in Fig. 1. After the update, 9795 registries were identified; after duplicates were removed, 4372 were screened; out of these, 762 met the screening criteria. However, 68 studies were not found, and the authors did not respond, leaving 694 for full reading selection of which 324 studies were included.

**Fig. 2:**
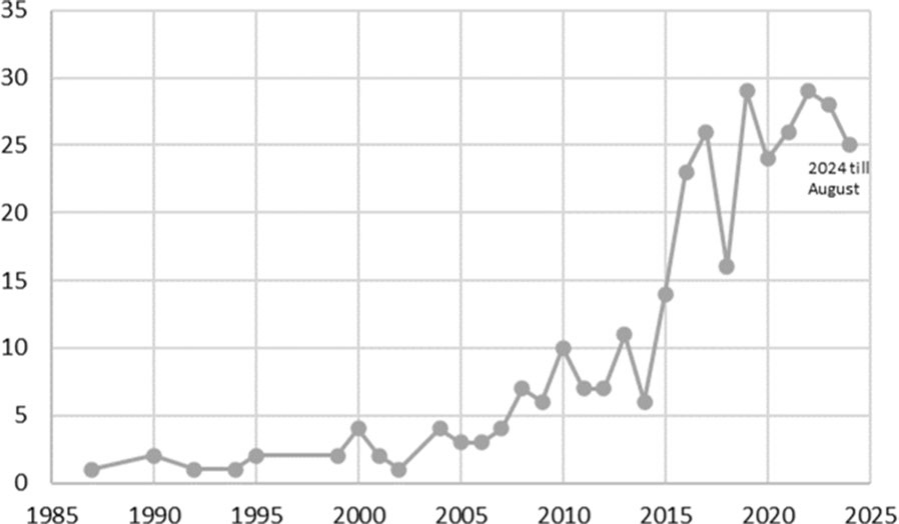
Publications per year

The list of the 324 studies included is provided in Additional File 1. Figure 2 indicates that the volume of studies on the theme increased considerably from 2015 onwards; of these publications included, 96% (312) were in the English language.

Table 1 presents the countries which most impacted the evaluation of their interventions: Australia (33 studies), United States (28), India (22), Brazil (16) and China (12). Table 2 indicates the countries of the institutions the first authors were affiliated with: United States (92), Australia (41), United Kingdom (29), Brazil (14) and Canada (14).

**Table 1:** Number of studies per country of intervention

Ranking	Country	*N*	%
1st	Australia	33	10
2nd	United States	28	9
3rd	India	22	7
4th	Brazil	16	5
5th	China	12	4
6th	Ghana	11	3
6th	Uganda	11	3
6th	World	11	3
9th	Mexico	10	3
9th	United Kingdom	10	3

**Table 2:** Number of studies per country of first author’s institution

Ranking	Country	*N*	%
1st	United States	92	28
2nd	Australia	41	13
3rd	United Kingdom	29	9
4th	Brazil	14	4
4th	Canada	14	4
6th	India	12	4
7th	Italy	11	3
8th	China	8	2
9th	Mexico	7	2
9th	Spain	7	2

The types of healthcare interventions being evaluated were mostly on the management of the hospital, government or health-related institution (35%); an example of this intervention is performance-based financing [14], followed by direct health assistance interventions (24%); educational health interventions (19%), that is, any learning or teaching related intervention; and health promotion (17%), including preventive campaigns and other communications. The least common types of interventions with included evaluations were research (3%) and pharmacologic (2%). The only specific design for the types of interventions mentioned was Kirkpatrick’s for education (5 out of 62 observed cases) [15].

Another result is whether the evaluation occurred before or after the intervention; all the included studies were ex-post. In addition, only six studies identified themselves as ex-post studies.

### Methodologies used in impact evaluations of healthcare interventions

Regarding the results on the methods used, firstly, purely quantitative approaches were dominant (81% of the studies), followed by mixed methods (10%), qualitative methods (6%) and reviews (3%). The studies were classified into five levels of comparison: strong counterfactual (58%); weak counterfactual (16%), that is, the ones with an effort to create a comparison group or econometric model but that are not strongly comparable considering the potential outcomes model [6]; comparison of pre and post only (15%); no comparison (7%); and some studies, such as reviews, for which counterfactuals do not apply (3%). Considering only the past 5 years, strong counterfactual designs comprised 63% of the total, a larger increase than the other comparison levels.

Out of the 324 studies, 120 were natural experiments or quasi-experimental designs (with strong counterfactual). Table 3 indicates the specific designs applied to these studies: difference-in-difference estimations; matching; or a combination of both, which represented 79% of all designs.

**Table 3:** Specific quasi-experimental designs found in the studies included

Design	*N*	Accumulated percentage
Differences-in-differences (diff-diff)	44	37
Diff-diff with matching	30	62
Matching (any kind)	21	79
Interrupted time-series	18	94
Instrumental variables	4	98
Regression discontinuity	2	99
Triple differences	1	100
Total	120	

### Available evidence

As for competing interests, 60% reported not having any, 28% did not report and 12% reported having some kind. As assessed by the authors of this research, in this theme, on the basis of the affiliations of the study’s authors, financing and execution of intervention, as well as evaluation, 28% seem to have some kind of conflict, whereas 72% do not seem to have any kind of competing interest. Another topic analysed is publication bias. On the basis of the results collected, 59% report fully positive results and up to 80% include mostly positive results. These data items were included to identify the evidence available as stated in the secondary research question.

Publication bias was crossed with conflict of interest and strength of comparison (Table 4), not having conflict of interest was 9 percentage points (p.p.) less associated with purely positive results, whereas the level of comparison evidence had even stronger effect, purely positive results were 80% present on only pre- and post-analysis, compared with 53% of the cases with strong counterfactual, that is, a 27 p.p. difference.

**Table 4:** Publication bias per counterfactual level of the studies included

	Strong (%)	Weak (%)	Only pre–post (%)
Positive	53	72	80
Partially positive	26	11	18
No effect	14	11	0
Mixed effects	3	4	2
Does not apply	3	2	0

**Fig. 3:**
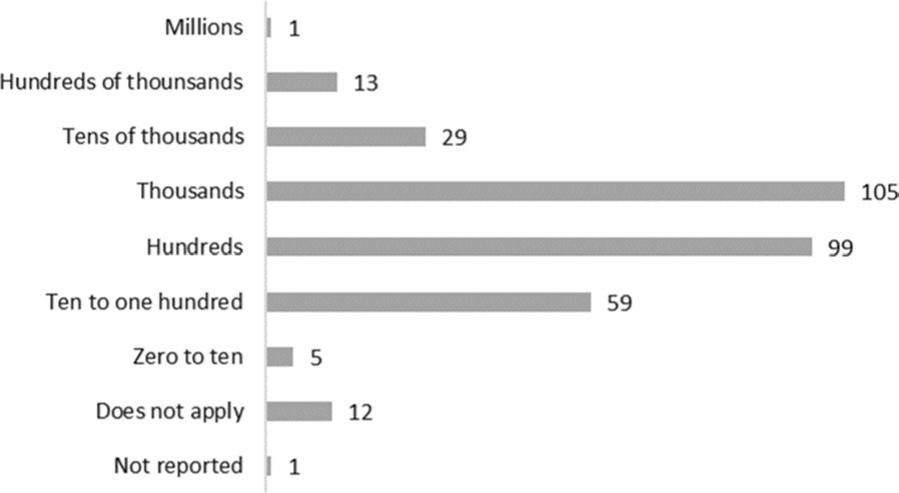
Number of observations in studies

Finally, Fig. 3 shows that the sample size of impact evaluations varied greatly, with a maximum sample of 3 435 068 observations from Pinto’s study compared with Robertson’s study, which had a sample size of 5 interviews [16, 17]. However, Pinto’s study was the only one included with a sample larger than a million observations. In total, 81% of the studies had a sample size between 10 and 9999 observations, making the median size 864.

## Discussion

The goal of this research was to map the methods associated with the impact evaluation term in the context of healthcare interventions, which has been increasing (Fig. 2). To the authors’ knowledge, there is no other scoping review of this term in general health interventions.

Guidelines such as those from Gertler and The Brazilian Ministry of Health are references for what is considered an impact evaluation of healthcare interventions [6, 8]. Although these guidelines point to studies that are ex-post, quantitative and with counterfactuals, such as Singh’s study on quality improvement for maternal and child health outcomes and Basinga and Gertler’s study on payment to primary care providers on the same type of population, qualitative studies, such as Edelman’s impact evaluation on research investment, were also expected [18–20]. However, guidelines mention ex-ante evaluations, and none were included in this scoping review.

As for specific nonexperimental, quantitative impact evaluation designs, although differences-in-differences and matching were very frequent, on the basis of representation in the guidelines, instrumental variables and discontinuity designs were expected to be more frequent than actually found (Table 3). This was the same for synthetic control designs, which appeared frequently during the selection phases, but none met the inclusion criteria; for example, Podestà’s study was not health-related and Abadie’s study on the California smoking program was not identified as an impact evaluation [21, 22].

In general, these results are aligned with reviews of impact evaluations of specific health interventions. Bardus’ scoping review on smoke-free policies in university settings identified all except one study as quantitative [23]. Degroote’s results from the study on health insurance in Sub-Saharan Africa [3] were 92% quantitative, whereas 56% had observational, 41% quasi-experimental and 3% experimental designs, also identifying an increase in quasi-experimental studies. Balancing Degroote’s results, Betts’ review on intervention in children with fetal alcohol spectrum disorder found six experimental, one quasi-experimental and four observational designs (pre–post) but the latter only included quantitative studies [24]. As for Colchero’s review of Mexico’s Seguro Popular, all 26 studies included were quasi-experimental designs, mostly matching, followed by instrumental variable models. In this latter review, the authors only included rigorous designs [10].

These results of exhaustive reviews on impact evaluations of specific healthcare interventions are also very much aligned with the guidelines previously mentioned. Particularly, the studies were predominantly quantitative and specifically used methods that identify the causality of the interventions, such as the experimental and quasi-experimental designs. The concordance of the results to these reviews answered the primary research question of this study on the methods used on the theme.

When inquiring about the available evidence, the secondary research question, the authors explored the integrity and transparency of publications. A noteworthy result of this research relates to two data items seeming to mitigate publication bias, that is, the reporting of only positive results. Although this is not an evidence of causality, by crossing variables in these results, both conflict of interest and having stronger comparison designs (counterfactuals) seem to be associated with less publication bias; however, the strongest designs had a much larger association (27 p.p.), as compared with having any conflict of interest (9 p.p.), as seen in Table 4. An example of study is Kruger’s evaluation of the Brazilian Integrated Border Health System. The authors used propensity score matching design (counterfactual approach), do not seem to have any conflict of interest and found no effect of the intervention (thus, no publication bias) [25]. The result of this scoping review not only stimulates further research but also has direct relevance to policymaking and policy evaluation, which require research integrity and transparency from these evaluations as seen in Kruger’s article.

As for using specific frameworks for specific kinds of interventions, this was only found in health-related educational interventions, which used Kirkpatrick’s model [15]. An example is White’s evaluation of the WHO safety checklist course [26]. However, this framework was only identified in 8% of the cases, suggesting that for the interventions mapped, there are no specific impact evaluation methods for particular types of interventions, but the general methods listed in this research.

However, a limitation of this scoping review refers to the data items chosen to be collected; for example, other categories for interventions, such as the International Classification of Health Interventions, would be relevant to cross with the impact methods mapped [27]. Furthermore, collecting variables reflecting epidemiological studies, health technology assessment and other public health-related categories could bring additional rich results.

Another relevant limitation of this study refers to terminology used during the identification and selection of the review when choosing studies that identified themselves as impact evaluations. Many well-known studies on health were not included because they did not explicitly identify themselves as such, for example, Abadie’s study [22]. The choice was made to include studies which would not be considered impact evaluations according to guidelines; nevertheless, they identified themselves as such. Consequently, many peer-reviewed impact evaluations of healthcare interventions were not on the list of the 324 studies included.

Finally, this review only categorized comparison groups within designs, leaving plenty of opportunity for further research into quality assessments of these studies. In addition, the present investigation demonstrates the increasing importance of impact evaluation of healthcare interventions, particularly natural experiments and quasi-experimental designs, opening the way to further explore this topic.

Overall, this scoping review fills a gap in the methods used to evaluate the impact of healthcare interventions, providing insights into the proportions they had on the theme.

## Conclusions

This research identified 324 studies that met the inclusion criteria. As expected, their methodologies vary widely, but most follow guidelines that suggest ex-post, quantitative (or mixed methods) with counterfactuals, that is, strong comparison. Furthermore, publications using these designs have been increasing in the past 5 years and, upon analysis, they not only seem to promote rigor in evaluation research but also integrity and transparency.

## Supplementary Information

Additional File 1. The list of studies included; full search strategies; inclusion criteria; and extraction data items.

## Data Availability

No datasets were generated or analysed during the current study.

## Abbreviations

WoS: Web of science
N: Number
%Acum: Accumulated percentage
Diff-diff: Differences-in-differences design
p.p.: Percentage points
